# Adaptive Zincophilic Synergistic Double-Network Hydrogel Electrolyte for Low-Temperature Long-Life Zinc Batteries

**DOI:** 10.3390/mi17060662

**Published:** 2026-05-27

**Authors:** Xiyao Huang, Wenwu Wang, Yibo Xiong, Zeyu Ma, Zilu Hu, Huimin Liang, Xiaoqiao Liao, Hongbin Su, Liang He, Xiaoyu Liu

**Affiliations:** 1 State Key Laboratory of Intelligent Construction and Healthy Operation and Maintenance of Deep Underground Engineering, School of Mechanical Engineering, Sichuan University, Chengdu 610065, China; huangxiyao111@stu.scu.edu.cn (X.H.); wangwenwu@stu.scu.edu.cn (W.W.); 2023223020040@stu.scu.edu.cn (Y.X.); mazeyu@stu.scu.edu.cn (Z.M.); huzilu@stu.scu.edu.cn (Z.H.); liang_huimin@stu.scu.edu.cn (H.L.); liaoxiaoqiao2@stu.scu.edu.cn (X.L.); m15095542960@163.com (H.S.); 2 Med+X Center for Manufacturing, West China Hospital, Sichuan University, Chengdu 610041, China; 3 School of Mechanical Engineering, Sichuan University, Chengdu 610065, China

**Keywords:** aqueous zinc-ion batteries, hydrogel electrolyte, wide-temperature range, solvation sheath reconstruction, flexible solid-state battery

## Abstract

Aqueous zinc-ion batteries are promising for large-scale energy storage due to their intrinsic safety, low cost, and environmental friendliness. However, their practical application is severely impeded by water-induced parasitic reactions and uncontrollable dendrite growth at the anode interface. Furthermore, the freezing of aqueous electrolytes at subzero temperature restricts their all-weather viability. Herein, we report a hydrogel electrolyte with interfacial regulation capabilities. By optimizing interfacial ion transport, the hydrogel electrolyte guides uniform Zn^2+^ deposition, effectively mitigating parasitic reactions and dendrite growth while enabling exceptional low-temperature tolerance. Consequently, the symmetric Zn//Zn cell using the hydrogel electrolyte delivers ultra-high cycling stability for 4000 h at 0.5 mA cm^−2^ under −30 °C. When assembled into full cells, the Zn//NH_4_V_4_O_10_ configuration operates stably for 4000 cycles at 5 A g^−1^, exhibiting outstanding capacity retention. Furthermore, the assembled flexible pouch cell maintains 86% of initial capacity after 900 cycles at 3 A g^−1^. Notably, the pouch cells demonstrate reliable operation and structural integrity under severe conditions, such as ice baths, bending, and piercing. This work provides an effective strategy for durable, wide-temperature, and intrinsically safe flexible aqueous energy storage systems.

## 1. Introduction

Driven by the escalating demand for cost-effective, safe, and sustainable energy storage systems, aqueous zinc-ion batteries (AZIBs) have emerged as formidable contenders for next-generation large-scale applications, owing to their high theoretical capacity (820 mAh g^−1^), low redox potential (−0.76 V vs. SHE), natural abundance, and inherent safety [1]. Nevertheless, their practical deployment is severely hindered by the thermodynamic instability of the zinc anode interface. Specifically, under aqueous environments, the active zinc surface undergoes unavoidable hydrogen evolution reactions (HER) and corrosion, accompanied by the formation of irreversible, insulating by-products (e.g., Zn_4_SO_4_(OH)_6_). These issues lead to compromised Coulombic efficiency and accelerated electrolyte depletion [2,3,4]. More critically, inhomogeneous zinc deposition triggers uncontrolled dendrite growth, which can penetrate the separator and cause cell failure through internal short-circuiting [5,6]. Furthermore, because of the intrinsic physicochemical properties of water, conventional aqueous electrolytes tend to freeze at subzero temperature, resulting in sluggish ion transport kinetics. This significantly limits the all-weather operational capability of AZIBs under extreme climatic conditions [7,8].

To counteract these dilemmas, quasi-solid-state hydrogel polymer electrolytes (HPEs) have garnered significant attention, leveraging their inherent mechanical flexibility, tunable molecular architectures, and superior water-retention capabilities. Beyond functioning as a robust physical barrier to suppress dendrite growth, HPEs effectively immobilize water molecules via polar functional groups on the polymer chains, thereby mitigating parasitic side reactions [9,10,11]. Nevertheless, the development of contemporary hydrogel electrolytes is frequently constrained by a formidable trade-off between mechanical robustness, ionic conductivity, and anti-freezing performance [10]. Conventional single-network (SN) hydrogels, such as those based on pure PAM or PVA, generally suffer from inadequate mechanical strength and poor water retention. These deficiencies render them vulnerable to penetration by sharp Zn dendrites and susceptible to structural collapse under external mechanical stresses, such as bending or compression [12]. Consequently, the construction of dual-network (DN) or multi-scale crosslinked architectures has emerged as a prevalent strategy [13,14,15]. By integrating rigid and flexible networks with dense physical entanglements, DN hydrogel electrolytes exhibit exceptional tensile toughness and compressive strength, which are instrumental in effectively suppressing the proliferation of zinc dendrites [16,17]. Nevertheless, their capacity for micro-level chemical regulation at the zinc anode interface remains highly restricted. Physical confinement fails to fundamentally eradicate the thermodynamic instability of water molecules at the anode interface [18].

To further mitigate parasitic reactions, it is imperative to reconstruct the Zn^2+^ solvation sheath at the molecular level and proactively construct a functional solid electrolyte interphase (SEI) layer on the zinc anode surface [19,20]. Indeed, advanced surface and phase engineering strategies, as well as the utilization of emerging 2D materials (e.g., MXenes and group-VA antimonene/bismuthene), have recently been demonstrated as highly effective pathways for advancing various next-generation energy storage and conversion systems [21,22,23]. Inspired by these rational interfacial tuning concepts, recently, water-in-salt electrolytes [24], organic co-solvents [25], and zincophilic molecular additives [26] have been successively proposed. These strategies aim to disrupt the hydrogen bonding network among free water molecules and diminish water activity, thereby suppressing interfacial parasitic reactions, while endowing the batteries with the ability to operate at low temperature. However, the elevated viscosity associated with these approaches severely compromise ion transport kinetics [27]. Furthermore, conventional hydrogels typically rely on physical contact with the electrodes, and lack strong interfacial chemical bonding, making them highly susceptible to interfacial delamination during prolonged cycling and leading to a drastic surge in interfacial impedance [28,29]. Therefore, developing a novel hydrogel electrolyte with high mechanical toughness, excellent ionic conductivity, exceptional interfacial affinity, and wide-temperature tolerance represents a viable pathway to address the current limitations of AZIBs.

Inspired by the multiple-interaction reinforcement mechanisms in biological tissues and the underwater adhesion chemistry of mussels, herein, we propose a multi-scale synergistic regulation strategy to construct a high-performance hydrogel electrolyte, from a poly(N-acryloylglycinamide)/polyacrylamide (PNAGA/PAM) dual-network matrix, coupled with dopamine (DA) interfacial modification and Zn(ClO_4_)_2_ solvation regulation. This composite hydrogel is designated as PNCD, where “PN” represents the PAM and NAGA polymer matrix, “C” stands for the ClO_4_^−^ anion, and “D” denotes DA. Within this architecture, the unique dual-amide motifs on the PNAGA side chains construct a pervasive hydrogen-bonding network. This structural feature endows the gel matrix with exceptional mechanical robustness, effectively suppressing dendrite proliferation [30,31,32]. Concurrently, acting as a typical Hofmeister anion, ClO_4_^−^ efficiently disrupts the long-range hydrogen-bonding network among water molecules. This disruption enables the electrolyte to resist freezing and maintain high ionic conductivity even at a subzero temperature of −30 °C [33,34]. Furthermore, the introduced DA not only facilitates the in situ formation of a SEI layer on the zinc anode to homogenize the ion flux but also leverages its catechol functional groups to establish chemical anchoring between the hydrogel and the electrode, thereby fundamentally ameliorating the issue of poor interfacial contact [35,36,37]. Benefiting from the synergistic interplay of these components in regulating mechanical strength, thermodynamics, and interfacial chemistry, the resultant composite hydrogel electrolyte exhibits exceptional electrochemical performance and wide-temperature adaptability. Consequently, the assembled Zn//Zn symmetric cells deliver remarkable cycling stability at both room temperature and −30 °C, achieving ultra-long lifespans of 2400 h and 4000 h at 0.5 mA cm^−2^, respectively. When coupled with an NH_4_V_4_O_10_ cathode, the assembled full cells maintain nearly their initial capacity after 4000 stable cycles at room temperature. Even at −30 °C, they retain a substantial capacity of 95 mAh g^−1^ after 2000 cycles. Moreover, the flexible pouch cells deliver a high capacity retention of 86% over 800 cycles. Overall, the PNCD hydrogel electrolyte demonstrates tremendous potential for the development of durable and flexible aqueous batteries tailored for extreme environments.

## 2. Materials and Methods

### 2.1. Materials

Acrylamide (AM), *N*-acryloyl glycinamide (NAGA), dopamine hydrochloride (DA), zinc perchlorate hexahydrate (Zn(ClO_4_)_2_·6H_2_O), ammonium metavanadate, and oxalic acid dihydrate were purchased from Aladdin Reagent Co., Ltd. (Shanghai, China). Ammonium persulfate (APS) and *N*,*N*′-methylenebisacrylamide (MBAA) were acquired from Chengdu Kelong Chemical Co., Ltd. (Chengdu, China).

### 2.2. Preparation of the PNCD Hydrogel Electrolyte

The PNCD hydrogel electrolyte was synthesized via a straightforward one-pot method. Initially, AM and NAGA monomers (total weight fraction of 22.5%) were dissolved in deionized water and stirred magnetically for 0.5 h to ensure homogeneity. Subsequently, 3 M Zn(ClO_4_)_2_·6H_2_O was added to the precursor and stirred until a clear solution was formed. Following this, DA (5 mM) was introduced into the mixture and stirred for another 0.5 h. Finally, APS (0.5 wt% relative to the total monomer mass) and MBAA (0.5 wt% relative to the total monomer mass) were added as the initiator and cross-linker, respectively. After thorough mixing, the resulting solution was transferred into a mold and polymerized in an oven at 60 °C for 8 h to obtain the PNCD hydrogel.

The PANA hydrogel (i.e., poly (acrylamide-co-*N*-acryloylglycinamide)) was prepared using the same procedure but with only AM and NAGA monomers. The 2 M ZnSO_4_ (Chengdu Kelong Chemical Co., Ltd., Chengdu, China) aqueous solution is termed as the ZSO (ZnSO_4_) electrolyte.

### 2.3. Preparation of the NH_4_V_4_O_10_ Cathode

The ammonium vanadate (NH_4_V_4_O_10_) was synthesized via a conventional hydrothermal method [38]. To prepare the cathode, NH_4_V_4_O_10_, conductive carbon (Super P, Shenzhen Kejing Star Technology Co., Ltd., Shenzhen, China), and polyvinylidene fluoride (PVDF, Shanghai Huayi 3F New Materials Co., Ltd., Shanghai, China) binder were mixed at a mass ratio of 7:2:1 in N-methyl-2-pyrrolidone (NMP, Chengdu Kelong Chemical Co., Ltd., Chengdu, China) solvent and stirred until a homogeneous slurry was formed. The resulting slurry was uniformly coated onto carbon cloth current collectors and subsequently dried in a vacuum oven at 60 °C. The typical mass loading of the active material on the cathode was approximately 2 mg cm^−2^.

### 2.4. Assembly of the Coin Cells

The CR2032 (Shenzhen Kejing Star Technology Co., Ltd., Shenzhen, China) coin full cells were fabricated using ammonium vanadate as the cathode, zinc foil as the anode, and PNCD as the electrolyte. The symmetric cells were assembled in the same manner, with both the cathode and anode being zinc foils. For the control groups, the electrolyte was replaced with PANA and ZSO.

### 2.5. Assembly of the Pouch Cells

The pouch cells were assembled using a sandwich structure. The zinc foil and the ammonium vanadate-loaded carbon cloth were cut into 4 × 4 cm^2^, while the PNCD was cut into 6 × 6 cm^2^. The flexible pouch cell was fabricated by pressing the zinc foil anode and the ammonium vanadate cathode onto opposite sides of the PNCD hydrogel electrolyte.

### 2.6. Material Characterizations

The cross-sections of the samples were observed using a scanning electron microscope (SEM, GeminiSEM 300, ZEISS, Oberkochen, Germany). Chemical composition analysis was performed utilizing Fourier transform infrared (FTIR, Bruker INVENIO R spectrometer, Bruker Optics, Ettlingen, Germany) spectroscopy and X-ray photoelectron spectroscopy (XPS, K-Alpha, Thermo Scientific, Waltham, MA, USA), while mechanical properties were tested using a universal testing machine. Thermal characteristics were investigated through differential scanning calorimetry (DSC, DSC Q2000, TA Instruments, New Castle, DE, USA) curves. The phase and morphology of the zinc surface after cycling were confirmed by X-ray diffraction (XRD, XRD-6100 diffractometer, Shimazu, Kyoto, Japan) and SEM, respectively.

### 2.7. Electrochemical Measurements

The linear sweep voltammetry (LSV) measurements were conducted using Zn//Zn symmetric coin cells at a scan rate of 1 mV s^−1^. The voltage sweep ranges were set from 0 to 3 V and from 0 to −1 V, respectively. The zinc-ion transference number (*t*_Zn_^2+^) was evaluated using Zn//Zn symmetric cells via the Bruce-Vincent method, which combines DC polarization and AC impedance measurements. The cell was thoroughly polarized by applying a constant DC voltage step (Δ*V* = 10 mV) to record the continuous chronoamperometry decay curve until a steady state was reached. Cyclic voltammetry tests (0.3 to 1.6 V) and electrochemical impedance spectroscopy (100,000 Hz to 0.01 Hz) were recorded on a CHI660E electrochemical workstation (Chenhua Instruments, Shanghai, China). Galvanostatic charge/discharge measurements of Zn//Zn symmetric cells were tested by the LAND battery testing system (CT3004A, Lanhe, Wuhan, China). The Coulombic efficiency of the Cu//Zn asymmetric cell is characterized by the LAND. The ionic conductivity of the PNCD hydrogel electrolyte was measured and calculated using alternating-current impedance (AC). The hydrogel was placed between two stainless steel electrodes, and the contact area (A) is 1.539 cm^2^. The thickness (L) of the tested hydrogel is 0.1 cm. The ionic conductivity was calculated by the equation: $\sigma =\frac{L}{R\cdot A}$, in which σ (S cm^−1^), L (cm), R (Ω), and A (cm^2^) are the ionic conductivity, thickness, the bulk resistance (intercept at Z’ axis in AC impedance spectra), and area.

## 3. Results and Discussion

**Figure 1:**
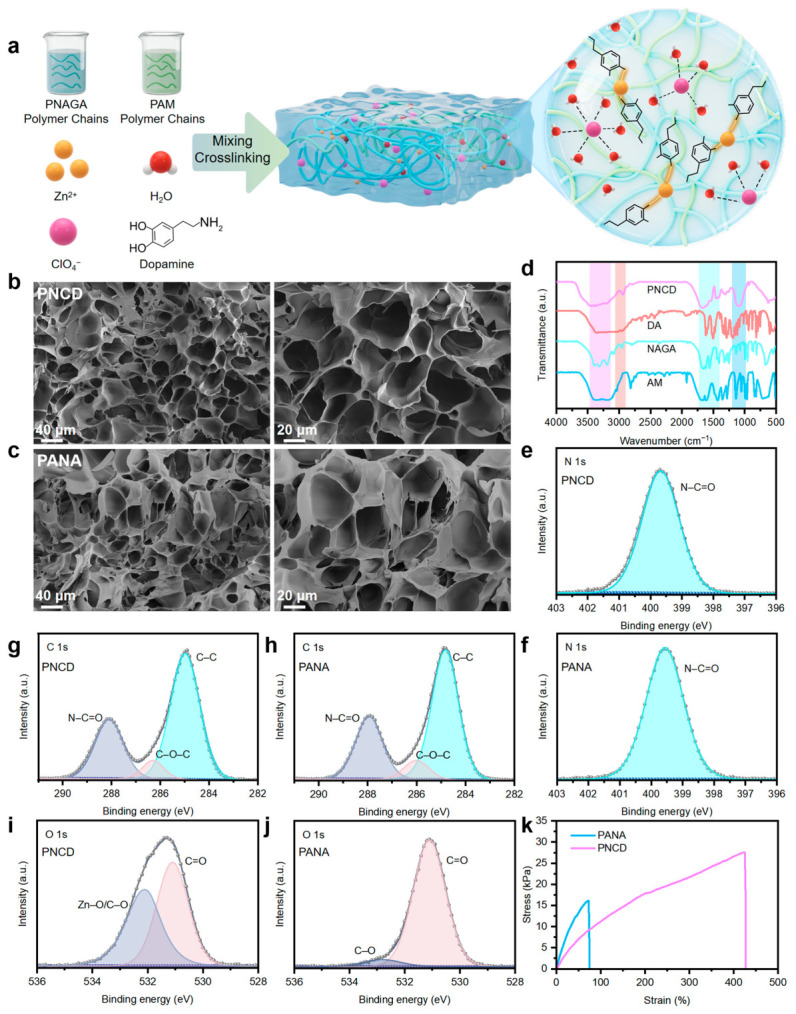
(**a**) Schematic diagram of the fabrication process and network structure of the PNCD. SEM images of (**b**) PNCD and (**c**) PANA. (**d**) FTIR spectra of PNCD, DA, NAGA, and AM. XPS N 1s spectra of (**e**) PNCD and (**f**) PANA. XPS C 1s spectra of (**g**) PNCD and (**h**) PANA. XPS O 1s spectra of (**i**) PNCD and (**j**) PANA. (**k**) Stress-strain curves of PNCD and PANA.

As illustrated in Figure 1a, the PNAGA and PAM were utilized to construct a robust dual-network polymer matrix. Within this crosslinked framework, the catechol and amino functional moieties of DA not only endow the hydrogel with superior interfacial affinity but also facilitate the formation of a dynamic molecular network via extensive hydrogen bonding and coordination interactions with both the polymer backbones and Zn^2+^. The SEM analysis of the lyophilized samples demonstrates that both the PANA and the modified PNCD hydrogels possess a characteristic 3D interconnected porous morphology. However, in contrast to the PANA (Figure 1c), the PNCD exhibits an evolved structure characterized by thinner pore walls and enlarged pore dimensions (Figure 1b). This highly open and interconnected 3D porous skeleton is instrumental in reducing ion transport resistance, thereby establishing unobstructed pathways for rapid Zn^2+^ migration [39].

To elucidate the chemical bonding and intermolecular interactions within the PNCD, FTIR spectroscopy was initially conducted (Figure 1d). The FTIR spectrum of the PNCD distinctly exhibits the characteristic absorption peaks of its constituent components. Specifically, the broad absorption band located in the 3200–3500 cm^−1^ is primarily ascribed to the overlapping signals of O–H stretching vibrations (originated from DA and residual water) and N–H stretching vibrations (derived from the amide monomers). The emergence of this characteristic band preliminarily verifies the successful incorporation of these functional moieties into the hydrogel. Furthermore, the intense absorption peak observed between 1600 and 1700 cm^−1^ corresponds to the classic amide I band, which predominantly originates from the C=O stretching vibrations of the amide linkages within the polymer backbone [40]. Compared with the pristine monomers, the peak profile of the PNCD in this region exhibits noticeable convolution and broadening. This phenomenon can be primarily attributed to the robust hydrogen-bonding interactions between the introduced dopamine molecules and the C=O groups of the polymer matrix. The extensive intermolecular hydrogen bonds create diverse vibrational states, and the superposition of these adjacent signals results in the observed convoluted peak profile. This further substantiates the successful copolymerization of DA within the dual-network polymer matrix. Concurrently, the saturated C–H stretching vibration peak located at 2900 cm^−1^ provides corroborative evidence for the completion of the thermal polymerization process. Moreover, the broad peak observed in the 1000–1200 cm^−1^ encompasses both the broadened C–N/C–O signals post-polymerization and the characteristic absorption of ClO_4_^−^, thereby confirming the effective integration of the inorganic zinc salt into the hydrogel matrix.

Further insights into the chemical environments were provided by XPS analysis. The N 1s spectra (Figure 1e,f) of both PNCD and PANA display a nearly identical main peak position at 399.5 eV. Concurrently, their C 1s spectra (Figure 1g,h) can be similarly deconvoluted into three characteristic peaks corresponding to C–C (284.8 eV), C–O–C (286.2 eV), and N–C=O (288.0 eV). These consistent features indicate that PNCD retains a polymer backbone architecture highly analogous to that of PANA. This structural similarity is further reflected in their O 1s spectra (Figure 1i,j), where both samples exhibit the characteristic C=O peak (532.5 eV). Nevertheless, the pivotal distinction at the 532.1 eV, where PNCD displays a drastically amplified peak intensity. This prominent peak is assigned to the convolution of Zn–O coordination bonds and C–O groups, directly corroborating the robust coordination interaction between the Zn^2+^ and the oxygen-containing moieties within the gel matrix. Such coordination interactions between oxygen atoms and zinc ions not only regulate the deposition kinetics of Zn^2+^ but also act as physical crosslinking junctions to reinforce network connectivity, thereby endowing the PNCD with superior mechanical robustness [41]. As illustrated by the stress-strain curves (Figure 1k), the PANA fractures at a relatively low tensile strain of ~75% with a maximum tensile stress of merely ~16 kPa, reflecting inadequate mechanical toughness. In contrast, the mechanical properties of the PNCD are substantially augmented. Its elongation at break surges to approximately 420%, accompanied by an elevated ultimate tensile stress of ~28 kPa. This structural reinforcement demonstrates the electrolyte’s exceptional resistance to tearing and deformation, which holds crucial practical value for withstanding internal physical punctures from zinc dendrites and external mechanical stresses during prolonged battery cycling.

**Figure 2:**
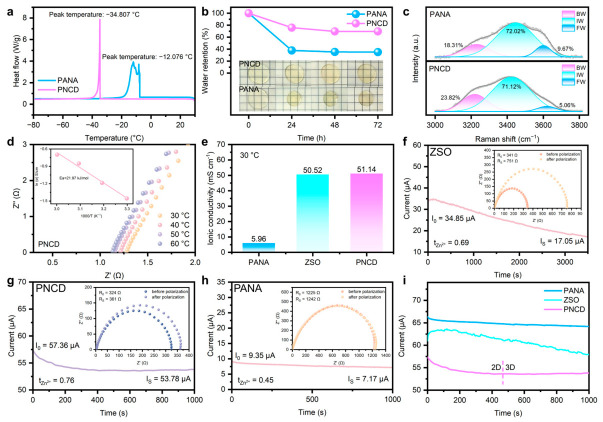
(**a**) DSC curves of PNCD and PANA. (**b**) Water retention rates of PNCD and PANA over time, and optical images of PNCD and PANA after standing at room temperature for 72 h. (**c**) Raman spectra of PNCD and PANA. (**d**) EIS spectra of PNCD at different temperatures and corresponding Arrhenius fitting plot. (**e**) Ionic conductivities of PNCD, ZSO, and PANA at 30 °C. Zinc-ion transference number of (**f**) ZSO, (**g**) PNCD, and (**h**) PANA (the insets show the impedance spectra before and after polarization). (**i**) CA curves of PNCD, ZSO, and PANA.

Owing to the abundance of free water, conventional hydrogels are highly prone to severe dehydration in open environments and rapid freezing at subzero temperature, inevitably leading to device failure. To evaluate the thermodynamic characteristics of the PNCD, DSC measurements were initially conducted. As shown in Figure 2a, the PANA exhibits a exothermic peak at −12.076 °C, corresponding to the crystallization phase transition of internal water molecules. In comparison, the freezing peak of the PNCD is significantly shifted to −34.807 °C. This pronounced freezing point depression is primarily attributed to a dual-synergistic mechanism. On the one hand, the ClO_4_^−^, acting as a typical Hofmeister anion, strongly disrupts the long-range tetrahedral hydrogen-bonding network among water molecules due to its low charge density, thereby thermodynamically elevating the phase-transition energy barrier for water crystallization [42]. On the other hand, the polar catechol and amino groups in DA, together with the abundant amide motifs on the polymer backbone, firmly anchor a massive amount of free water via dense hydrogen-bonding interactions, converting it into non-crystallizable bound water [43]. This potent molecular-level “water-locking” effect is intuitively corroborated by macroscopic water retention tests. As depicted in Figure 2b, after standing at room temperature for 72 h, the water retention of the PANA sharply plummets to 38%, accompanied by severe macroscopic volume shrinkage and desiccation. In contrast, the PNCD not only maintains a high water retention of 71%, but also exhibits only slight morphological or dimensional variations in the optical photographs, persistently remaining flexible state. These empirical results demonstrate that the molecular network engineered within PNCD effectively restrains the evaporation of liquid water, underscoring the potential of PNCD-based zinc-ion batteries to deliver stable, long-term operation under wide-temperature conditions.

To reveal this “water-locking” effect at the molecular level, Raman spectroscopy was further employed to probe the hydrogen-bonding states of water within the hydrogels. By deconvoluting the broad O–H stretching band (3000–3800 cm^−1^), water molecules can be classified into strongly hydrogen-bonded water (~3240 cm^−1^), intermediate water (~3430 cm^−1^), and free water (~3610 cm^−1^). As shown in Figure 2c, compared with the PANA, the PNCD hydrogel exhibits a substantial decrease in the relative intensity of the free water peak, accompanied by a notable increase in the strongly hydrogen-bonded water. This spectroscopic evidence confirms that the polar groups within the PNCD matrix effectively restrict free water into bound states, decreasing its thermodynamic activity and mitigating interfacial parasitic reactions.

To deeply explore the mechanism by which PNCD hydrogel electrolyte affects the zinc deposition/stripping process, we systematically compared its electrochemical behavior with ZSO electrolyte and PANA hydrogel electrolyte. Electrochemical impedance spectroscopy (EIS, Figure 2d) were conducted to evaluate the ion transport capabilities of the hydrogel electrolyte. Based on the Arrhenius fitting, the activation energy (*E_a_*) of the PNCD hydrogel was calculated to be 21.97 kJ/mol. Benefiting from the highly interconnected porous network within PNCD, the diffusion resistance of zinc ions across the polymer matrix is significantly lowered [44]. Consequently, as depicted in Figure 2e, the PNCD delivers an exceptional ionic conductivity of 51.14 mS cm^−1^ at 30 °C.

Beyond high ionic conductivity, the zinc-ion transference number (*t*_Zn_^2+^) serves as another pivotal descriptor for evaluating an electrolyte’s capability to mitigate concentration polarization and prevent dendrite proliferation. As illustrated in Figure 2f–h, the *t*_Zn_^2+^ for the ZSO electrolyte is measured as 0.69 via the potentiostatic polarization method, whereas the value for the PANA electrolyte is merely 0.45. Remarkably, the *t*_Zn_^2+^ of the PNCD hydrogel electrolyte is measured as 0.76. This substantial increase indicates that charge transport within the PNCD hydrogel electrolyte is predominantly mediated by the migration of Zn^2+^, while the free movement of anions is effectively immobilized. Such a homogenized and efficient zinc-ion flux is conducive to optimizing the deposition kinetics at the zinc anode, thereby providing favorable conditions for highly reversible zinc plating/stripping processes [45]. The transient current response from the chronoamperometry (CA) curves (Figure 2i) further elucidates this behavior. Under a constant overpotential, both the ZSO and the PANA electrolyte exhibit a continuous current decay over time, reflecting restricted interfacial diffusion kinetics and the uncontrolled nature of zinc deposition [46]. While the PANA hydrogel slightly mitigates this severe decay due to its basic physical confinement, its current still maintains a continuous downward trend throughout the testing period, indicating that a true dynamic equilibrium has not been reached. In contrast, the PNCD hydrogel electrolyte demonstrates a distinct steady-state characteristic: after an initial current drop corresponding to the interfacial 2D diffusion process, the curve rapidly stabilizes after approximately 450 s and maintains a constant current level for an extended period, indicating a successful transition to the 3D steady-state deposition stage. This smooth evolution from 2D to 3D steady-state diffusion directly confirms that the PNCD hydrogel network effectively homogenizes the interfacial ion flux and guides the zinc ions toward dense, regulated, and uniform 3D deposition on the anode surface [12].

**Figure 3:**
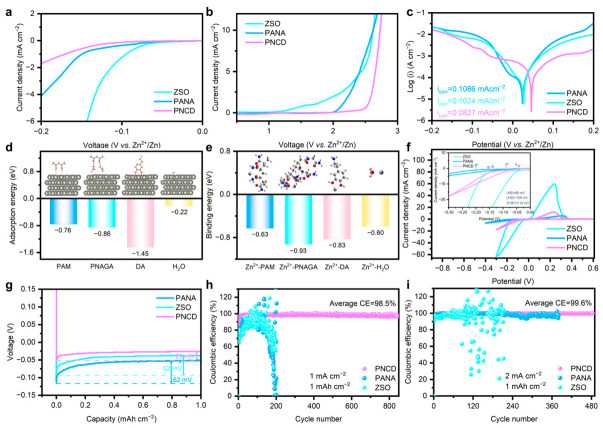
(**a**,**b**) LSV curves. (**c**) Tafel curves. (**d**) Adsorption energies of PAM, PNAGA, DA, and H_2_O on the Zn (002) crystal plane. (**e**) Binding energies of PAM, PNAGA, DA, and H_2_O with Zn^2+^. (**f**) CV curves of the Zn//Cu cell at a scan rate of 1 mV s^−1^ (the inset shows the nucleation overpotential). (**g**) Nucleation overpotential. Coulombic efficiency of the Zn//Cu cell at (**h**) 1 mA cm^−2^ and 1 mAh cm^−2^, and (**i**) 2 mA cm^−2^ and 1 mAh cm^−2^.

In AZIBs, the primary drivers of device failure reside in the vigorous water-induced parasitic reactions and the uncontrolled proliferation of Zn dendrites at the anode/electrolyte interface. To evaluate the efficacy of the PNCD hydrogel electrolyte in mitigating these interfacial side reactions, the electrochemical stability window (ESW) was first investigated via linear sweep voltammetry (LSV). As shown in Figure 3a,b, compared with both the ZSO and the PANA, the PNCD hydrogel electrolyte significantly broadens the voltage window by suppressing the hydrogen evolution reaction (HER) and oxygen evolution reaction (OER). This is primarily attributed to the sequestration of water molecules through intensive hydrogen-bonding interactions within the hydrogel network, which effectively diminishes the activity of free water. This superior interfacial stability is further corroborated by the Tafel polarization curves (Figure 3c). Remarkably, the corrosion current density (*i_corr_*) of the PNCD hydrogel electrolyte decreases to 0.0827 mA cm^−2^, substantially lower than those of ZSO (0.1024 mA cm^−2^) and PANA (0.1086 mA cm^−2^). These results demonstrate that the PNCD hydrogel electrolyte successfully inhibits parasitic side reactions on the zinc anode surface and bolsters the overall corrosion resistance [47].

To elucidate this interfacial protection mechanism at the molecular scale, density functional theory (DFT) calculations were performed (Figure 3d). The results reveal that the adsorption energy of DA on the Zn (002) crystal plane is −1.45 eV, exhibiting significantly higher thermodynamic spontaneity compared with the polymer components (PNAGA: −0.86 eV, PAM: −0.76 eV) and water molecules (−0.22 eV). This energetic hierarchy suggests that DA molecules preferentially anchor onto the zinc anode surface within the complex PNCD hydrogel. In close synergy with the PNAGA-PAM dual-network matrix, DA facilitates the in situ construction of a continuous protective network that integrates zincophilicity with physical shielding [46]. This hybrid interphase, jointly formed by the gel matrix and functional molecules, effectively minimizes direct contact between free water and the active zinc, thermodynamically suppressing parasitic reactions such as hydrogen evolution and corrosion. Beyond physical passivation, the PNCD hydrogel electrolyte participates in the reconstruction of the primary Zn^2+^ solvation sheath, thereby enhancing zinc deposition kinetics. DFT binding energy calculations (Figure 3e) confirm that the affinity of PNAGA segments (−0.93 eV) and DA molecules (−0.83 eV) for Zn^2+^ significantly exceeds that of water molecules (−0.60 eV). Correlated with the aforementioned XPS results, it can be inferred that the polar amide groups on the polymer backbone and the dopamine molecules collaboratively create a coordination-rich local microenvironment. These moieties synergistically compete with and effectively displace a portion of the free water molecules in the primary Zn^2+^ solvation shell. This reconstruction not only diminishes the local water activity but also substantially lowers the desolvation energy barrier for Zn^2+^ during interfacial charge transfer [48].

As depicted in Figure 3f,g, the nucleation overpotential (NOP) of the cell is substantially reduced. Specifically, the PNCD hydrogel electrolyte exhibits a remarkably low NOP of 23 mV, significantly lower than those of the ZSO (52 mV) and PANA (62 mV). Such a diminished NOP is instrumental in mitigating the localized tip-driven growth of dendrites, thereby promoting the uniform deposition of Zn^2+^ [49]. Benefiting from the suppression of interfacial parasitic reactions and the optimization of nucleation kinetics, the PNCD-based Zn//Cu cell demonstrates exceptional long-term reversibility in the plating/stripping cycling. As illustrated in Figure 3h, under the testing conditions of 1 mA cm^−2^ and 1 mAh cm^−2^, the Zn//Cu cells based on ZSO and PANA suffer from severe, uncontrolled dendrite proliferation and the accumulation of “dead zinc”. Consequently, their Coulombic efficiency (CE) exhibits drastic fluctuations within fewer than 200 cycles, ultimately leading to cell failure. In contrast, the PNCD-based Zn//Cu cell achieves highly stable cycling for over 800 cycles, establishing a steady-state equilibrium for the zinc plating/stripping processes on the copper substrate, delivering a high average CE of 98.5%. Even under more stringent conditions with elevated current densities (2 mA cm^−2^, Figure 3i), the PNCD-based Zn//Cu cell steadfastly maintains its robust cycling stability. These results unequivocally corroborate its excellent capability in enhancing the electrochemical reversibility of the zinc anode.

**Figure 4:**
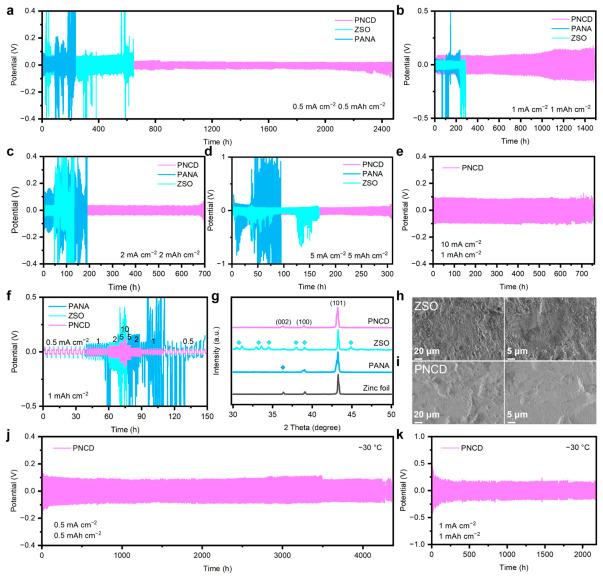
Cycling performance of the Zn//Zn cell at (**a**) 0.5 mA cm^−2^ and 0.5 mAh cm^−2^, (**b**) 1 mA cm^−2^ and 1 mAh cm^−2^, (**c**) 2 mA cm^−2^ and 2 mAh cm^−2^, (**d**) 5 mA cm^−2^ and 5 mAh cm^−2^, and (**e**) 10 mA cm^−2^ and 1 mAh cm^−2^. (**f**) Rate performance of the Zn//Zn cell at current densities of 0.5–10 mA cm^−2^ and a constant capacity of 1 mAh cm^−2^. (**g**) XRD patterns of the zinc anode after cycling for 100 h using different electrolytes and the pristine zinc foil. SEM images of the zinc anode surface after cycling for 100 h using (**h**) ZSO and (**i**) PNCD. Cycling performance of the Zn//Zn cell at −30 °C at (**j**) 0.5 mA cm^−2^ and 0.5 mAh cm^−2^, and (**k**) 1 mA cm^−2^ and 1 mAh cm^−2^.

To further evaluate the long-term interfacial stability and dynamic cycling performance of the zinc anode under practical operating conditions, symmetric Zn//Zn cells were assembled and subjected to galvanostatic charge/discharge testing. As illustrated in Figure 4a, under a current density of 0.5 mA cm^−2^ and an areal capacity of 0.5 mAh cm^−2^, the symmetric cells utilizing ZSO and PANA at room temperature exhibit a gradual escalation in polarization voltage. These cells undergo premature short-circuit failure induced by uncontrolled dendrite penetration through the separator within a limited lifespan. In contrast, benefiting from the suppression of interfacial parasitic reactions and the uniform 3D nucleation mechanism, the PNCD-based symmetric cells demonstrate remarkable cycling stability. They achieve an ultra-long, steady operation for 2400 h while maintaining a consistently low polarization overpotential. When subjected to higher testing current of 1 mA cm^−2^ and 1 mAh cm^−2^ (Figure 4b), the PNCD-based symmetric cell sustains a stable cycling lifespan of 1400 h. Furthermore, at substantially higher current densities (e.g., 2, 5, and up to 10 mA cm^−2^, Figure 4c–e), the PNCD-based symmetric cells persistently exhibit highly reliable electrochemical durability. The rate-capability testing (Figure 4f) further validates the robust ion transport kinetics of the cell. Under step-wise current densities ranging from 0.5 to 10 mA cm^−2^, the symmetric Zn//Zn cell based on the PNCD hydrogel electrolyte remains steady cycling performance. Notably, upon reverting the current density to 0.5 mA cm^−2^, the polarization voltage instantaneously recovers to its initial baseline. This rapid recovery unequivocally demonstrates that the hydrogel network preserves intact structural integrity and unobstructed transport pathways even under intense ion shuttling conditions.

The contrast in the long-term cycling lifespans of the symmetric cells can be rationally elucidated by the structural and morphological evolution of the zinc anode surfaces post-cycling. XRD (Figure 4g) analysis reveals that on the surface of the electrode cycled in ZSO electrolyte, alongside the characteristic diffraction peaks of the zinc substrate, several weak parasitic signals exist. This phenomenon primarily originates from the inevitable parasitic reactions inherent in aqueous batteries, which trigger localized interfacial pH fluctuations, consequently driving the sluggish accumulation of trace irreversible by-products (e.g., non-stoichiometric oxides or basic salts) during cycling [50]. Conversely, the XRD pattern of the zinc anode cycled in the PNCD-based symmetric cell aligns remarkably well with that of the pristine zinc foil, devoid of any discernible by-product signals. The interfacial protective layer engineered by the PNCD hydrogel electrolyte successfully shields the anode from water-induced degradation at the thermodynamic level, thoroughly suppressing parasitic reactions. This conclusion is visually corroborated by SEM images. As shown in Figure 4h,i, the zinc surface cycled in the PNCD hydrogel electrolyte presents a smooth, highly compact, and dendrite-free deposition morphology. In contrast, the zinc surface cycled in ZSO electrolyte appears excessively rough and porous, riddled with chaotic dendrites and accumulated “dead zinc”. The corroboration of structural and morphological analyses unambiguously validates the exceptional efficacy of the PNCD hydrogel electrolyte in guiding uniform zinc deposition and mitigating anodic corrosion.

More importantly, this molecular-level “water-locking” micro-mechanism similarly endows AZIBs with extraordinary low-temperature performance. Driven by the hydrogen-bond disruption of ClO_4_^−^ and the water-anchoring effect of the gel skeleton, the PNCD hydrogel electrolyte effectively averts freezing and the stagnation of ion transport at subzero temperature. As shown in Figure 4j,k, under the harsh condition of −30 °C, the PNCD-assembled symmetric cells achieve an ultra-long cycling lifespan of over 4000 h at 0.5 mA cm^−2^, and stably operate for over 2000 h at 1 mA cm^−2^. This exceptional performance at low temperature demonstrates that the cell can not only regulate interfacial chemical behaviors at room temperature, but also maintain stable charge-transfer kinetics at low temperature, providing strong experimental support for the development of wide-temperature aqueous flexible energy storage devices.

**Figure 5:**
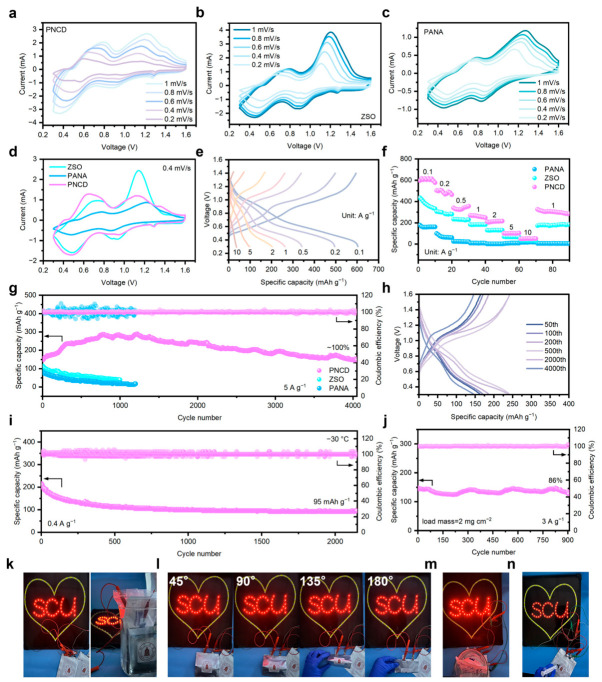
CV curves of the Zn//NH_4_V_4_O_10_ cell at different scan rates using (**a**) PNCD, (**b**) ZSO, and (**c**) PANA. (**d**) CV curves of the Zn//NH_4_V_4_O_10_ cell at 0.4 mV s^−1^. (**e**) Charge-discharge curves of the Zn//PNCD//NH_4_V_4_O_10_ cell at different rates. (**f**) Rate performance of the Zn//NH_4_V_4_O_10_ cell. (**g**) Cycling performance of the Zn//NH_4_V_4_O_10_ cell at 5 A g^−1^. (**h**) Charge-discharge curves of the Zn//PNCD//NH_4_V_4_O_10_ cell at different cycles. (**i**) Cycling performance of the Zn//PNCD//NH_4_V_4_O_10_ cell at −30 °C and 0.4 A g^−1^. (**j**) Cycling performance of the Zn//PNCD//NH_4_V_4_O_10_ pouch cell at 3 A g^−1^. (**k**) Digital photographs of two pouch cells connected in series powering an LED sign in a room temperature and in an ice bath. (**l**) Digital photographs of the pouch cell powering an LED sign under different bending angles (45°, 90°, 135°, and 180°). Digital photographs of the pouch cell powering an LED sign under (**m**) pressing and (**n**) piercing.

To evaluate the practical viability of the PNCD hydrogel electrolyte in integrated energy storage systems, Zn//NH_4_V_4_O_10_ full cells were assembled and subjected to systematic electrochemical characterization. Initially, the kinetic behavior of the full cells was explored via cyclic voltammetry (CV) at various scan rates (Figure 5a–c). As the scan rate increasing from 0.2 to 1.0 mV s^−1^, the redox peaks of the full cell exhibit minor polarization shifts. The overall CV profiles and primary peak positions remain highly consistent across all Zn//NH_4_V_4_O_10_ full cells using three electrolytes, confirming a uniform Zn^2+^ intercalation/deintercalation mechanism [51]. Notably, at a scan rate of 0.4 mV s^−1^ (Figure 5d), the PNCD-based full cell demonstrates a smaller potential difference (Δ*E*) between redox peaks compared with the ZSO counterpart, reflecting diminished electrochemical polarization resistance at the cell interface. Furthermore, it is noteworthy that the PNCD-based electrode exhibits a significantly higher current contribution in the low-potential region (<0.9 V vs. Zn^2+^/Zn). The charge storage typically involves a potential-driven selective co-insertion of H^+^ and Zn^2+^, where the low-potential region is predominantly governed by Zn^2+^ intercalation [52,53]. In the ZSO electrolyte, this process is kinetically sluggish due to the high desolvation energy barrier of the heavily hydrated Zn^2+^. However, the PNCD hydrogel electrolyte optimizes the interfacial solvation microenvironment and significantly lowers the desolvation energy barrier. This kinetically facilitates the deep insertion of Zn^2+^ into the cathode lattice, effectively activating the low-potential redox sites and unlocking superior capacity output. Rate capability tests (Figure 5e,f) further reveal that the PNCD-based full cell maintains responsive capacities even as the current density increasing from 0.1 to 10 A g^−1^. Upon returning the current density to 1 A g^−1^, the specific capacity promptly recovers to its initial baseline, underscoring the cell’s exceptional kinetic reversibility across a wide range of operating rates.

In the long-term cycling stability evaluation (5 A g^−1^, Figure 5g), the PNCD-based full cell exhibits the characteristic electrochemical evolution typical of vanadium-based cathodes. During the initial cycles, the cell capacity undergoes a distinct activation phase, followed by a gradual stabilization. This phenomenon is primarily attributed to the progressive infiltration of the electrolyte into the cathode, as well as the electrochemical oxidation of low-valence vanadium or microstructural rearrangements [54]. Remarkably, after an extensive 4000-cycle test, while the Zn//NH_4_V_4_O_10_ full cells using ZSO and PANA suffer from premature failure due to irreversible dendrite growth or interfacial side reactions, the PNCD-based cell sustains exceptionally stable operation, retaining nearly 100% of its initial specific capacity. The galvanostatic charge-discharge (GCD) profiles (Figure 5h) from the 50th to the 4000th cycle further corroborate the minimal polarization shift, underscoring the efficacy of the dual-network hydrogel in significantly extending the service life of the full cell. This superior stability originates from a dual-stabilization mechanism: the PNCD hydrogel electrolyte not only regulates Zn^2+^ deposition to suppress dendrite formation at the anode but also effectively inhibits the dissolution of ammonium vanadate moieties from the cathode, thereby comprehensively bolstering the electrochemical performance.

Equally compelling is the subzero energy storage performance of the full cell. Under the rigorous condition of −30 °C (Figure 5i), the PNCD-based full cell delivers a reversible specific capacity of ~95 mAh g^−1^ even after prolonged cycling for over 2000 cycles. In contrast to conventional aqueous batteries, which ubiquitously suffer from severe capacity degradation and premature failure at low temperature [55], the Zn//NH_4_V_4_O_10_ full cell based on the PNCD hydrogel electrolyte demonstrates robust electrochemical cyclability under low temperature. This resilient low-temperature operation further validates the feasibility of the hydrogel electrolyte for practical all-weather energy storage applications.

Finally, to validate the practical feasibility of this hydrogel electrolyte in flexible wearable electronics, integrated pouch cells were assembled. As shown in Figure 5j, the pouch cell, tested at a current density of 3 A g^−1^ (mass loading of ~2 mg cm^−2^), maintains 86% of initial capacity after 900 cycles with CE near 100%. In practical power supply demonstrations, two series-connected pouch cells successfully power an LED sign spelling “SCU” (consisting of dozens of LEDs) and maintain stable operation even when submerged in an ice bath (Figure 5k). This remarkable environmental and mechanical tolerance benefits from the polymer dual-network’s exceptional water retention, anti-freezing, and robust mechanical properties. Furthermore, it provides stable power output even when subjected to dynamic bending/folding (from 45° to 180°, Figure 5l), pressing (Figure 5m), and piercing (Figure 5n). This unequivocally confirms that the flexible solid-state battery based on the PNCD hydrogel electrolyte possesses ample commercial potential in extreme mechanical deformation and complex environments.

## 4. Conclusions

In summary, a NAGA-AM dual-network hydrogel electrolyte (PNCD) was developed through the synergistic integration of interfacial DA modification and Hofmeister anion regulation, effectively resolving the interfacial degradation and temperature-dependent failure of aqueous zinc anodes. Mechanistic investigations reveal that the polar polymeric network and DA cooperatively reconstruct the Zn^2+^ solvation sheath. This reconstruction optimizes the interfacial deposition kinetics and substantially suppresses parasitic hydrogen evolution, anodic corrosion, and dendrite proliferation. Furthermore, the synergistic coupling of ClO_4_^−^-induced hydrogen-bond disruption and the gel matrix’s “water-locking” confinement endows the electrolyte with exceptional anti-freezing capability, depressing the freezing point to −34.8 °C. Consequently, the PNCD-based symmetric cells deliver dendrite-free, ultra-long cycling lifespans of 2400 h at ambient temperature and 4000 h under −30 °C. The Zn//NH_4_V_4_O_10_ full cell demonstrates robust durability, operating stably for 4000 cycles at 5 A g^−1^ and sustaining 2000 cycles with a reversible capacity of 95 mAh g^−1^ even under −30 °C. For practical deployment, the integrated flexible pouch cell retains 86% of its initial capacity after 900 cycles at 3 A g^−1^. Crucially, it maintains structural integrity and provides uninterrupted power output even when subjected to severe mechanical abuse, including 180° folding, pressing, and piercing. By incorporating multifunctional moieties and specific salt ions into a dual-network matrix, this multi-dimensional synergistic strategy establishes a highly viable design paradigm for intrinsically safe, all-weather flexible aqueous energy storage systems.

## Data Availability

The original contributions presented in this study are included in the article. Further inquiries can be directed to the corresponding authors.
